# Total Salvianolic Acid Balances Brain Functional Network Topology in Rat Hippocampi Overexpressing miR-30e

**DOI:** 10.3389/fnins.2018.00448

**Published:** 2018-07-05

**Authors:** Qi Li, Liang Wang, Xin-Yi Li, Xiao Chen, Bin Lu, Long Cheng, Chao-Gan Yan, Yong Xu

**Affiliations:** ^1^Drug Clinical Trial Institution, Taiyuan Center Hospital of Shanxi Medical University, Taiyuan, China; ^2^Department of Psychiatry, First Hospital/First Clinical Medical College of Shanxi Medical University, Taiyuan, China; ^3^Shanxi Province Mental Health Center/Taiyuan Psychiatric Hospital, Taiyuan, China; ^4^Department of Neurology, Shanxi DaYi Hospital, Taiyuan, China; ^5^Institute of Psychology, Chinese Academy of Sciences, Beijing, China; ^6^MDT Center for Cognitive Impairment and Sleep Disorders, First Hospital of Shanxi Medical University, Taiyuan, China; ^7^National Key Disciplines, Key Laboratory for Cellular Physiology of Ministry of Education, Department of Neurobiology, Shanxi Medical University, Taiyuan, China

**Keywords:** miR-30e, memory dysfunction, RS-fMRI, graph theory, hippocampus

## Abstract

We investigated the therapeutic effects and underlying brain functional network topology mechanisms of total salvianolic acid (TSA) treatment for memory dysfunction by using miR-30e overexpression-induced memory deficit in rat hippocampi. Model rats were developed by lentivirus vectors carrying miR-30e into bilateral hippocampus CA1 region through stereo-surgery. Two weeks after surgery, TSA (20 or 10 mg/mL/kg) or saline were administrated for 14 consecutive days. Memory function was assessed by behavioral tests (Y maze and Morris water maze [MWM]); resting-state functional MRI (RS-fMRI); and molecular alterations of BCL-2, UBC9, and Caspase-3 in the hippocampus CA1 region, as detected by immunohistochemistry. Compared to controls, model rats exhibited significantly impaired working and long-term memory in the Y maze and MWM tests (*p* < 0.01). The brain functional network topology analyzed based on RS-fMRI data demonstrated that miR-30e disturbed the global integration and segregation balance of the brain (*p* < 0.01), and reduced edge strength between CA1 and the posterior cingulate, temporal lobe, and thalamus (*p* < 0.05, false discovery rate corrected). At the molecular level, BCL-2 and UBC9 were downregulated, while Caspase-3 was upregulated (*p* < 0.01). After TSA (20 mg/mL/kg) treatment, the biomarkers for behavioral performance, global integration and segregation, edge strength, and expression levels of BCL-2, UBC9, and Caspase3 returned to normal levels. The correlation analyses of these results showed that global brain functional network topologic parameters can be intermediate biomarkers correlated with both behavioral changes and molecular alterations. This indicated that the effects of TSA were achieved by inhibiting apoptosis of CA1 neurons to improve global functional network topology.

## Introduction

MicroRNAs (miRNAs) are endogenous approximately 22nt RNAs that participate in many physiological activities or pathological process by targeting mRNAs for cleavage or translational repression (Bartel, 2004). Increasing evidence has demonstrated that dysregulation of miRNAs affected mental process and were associated to various neuropsychiatric disorders(Sharma and Lu, 2018; Sun et al., 2018). Among these miRNAs, recent investigations implied that miR-30e may have complex effects. Previous researches indicated that miR-30e was an oncogenic miRNA in lung adenocarcinoma (Zhuang et al., 2017) or tumor suppressing miRNA in breast cancer (Liu et al., 2017), but in central nervous system miR-30e influenced many mental process and correlated to many psychiatric disorders, such as schizophrenia (Xu et al., 2010), cognitive impairment (Labouesse et al., 2018) and vulnerability to stress (Pearson-Leary et al., 2017). Our previous results also indicated that miR-30e overexpression in rat hippocampus could induce learning and memory impairment (Xu et al., 2015). Therefore, a miR-30e-induced memory deficit model was chosen to explore the therapeutic strategy of memory dysfunctions.

Total salvianolic acid (TSA) is an injectable solution of the major water-soluble ingredients extracted from *Salvia miltiorrhiza*, including 3, 4-dihydroxy-phenyl lactic acid, salvianolic acid A, salvianolic acid B, and other salvianolic acids, which can ameliorate the microcirculation disturbance induced by ischemia-reperfusion in rats (Wang et al., 2010). Further investigation suggests that TSA can bring about anti-oxidation and neuron protection via inactivation of NADPH oxidase through a signaling pathway implicating AMP-activated protein kinase (AMPK)/Akt/protein kinase C (PKC) (Tang et al., 2014). Besides the microcirculation protection effects of TSA, a recent study reported that TSA showed a remarkable improvement in learning and memory impairment in APPswe/PS1dE9 mice (Shen et al., 2017). However, the mechanisms that underline the memory protection effects of TSA are still unclear.

To evaluate curative effects of therapeutic strategies, many investigations mainly acquired data by behavioral test and molecular biomarkers (Sun et al., 2015; Wan et al., 2015). However, there is always a gap existing between behavioral changes and molecular alterations. To address this concern, magnetic resonance imaging (MRI), especially resting-state functional MRI (RS-fMRI), has been applied as a valuable translational tool to investigate alterations in brain structure and function in both patients and animal models of disease (McIntosh et al., 2017). RS-fMRI has recently been highlighted as a new technique for functional connectivity changes in subjects with different subtypes of mild cognitive impairment (MCI) and different stages of cognitive impairment, which avoids variability in task fMRI (Joo et al., 2016; Li et al., 2017; Pan et al., 2017). Similar findings were obtained in animal models, as resting-state functional connectivity data supports the detection of cognition in the rodent brain (Nasrallah et al., 2016). These findings were mainly acquired by independent component analysis or seed-based approaches to delineate functionally connected brain regions or networks of regions. Recently, graph theoretical methods have been applied to neuroimaging data in an attempt to obtain complementary measures on brain topology that will reflect brain organization on a more global level (Hadley et al., 2016). Therefore, the present study attempted to apply a multiscale analysis combined with brain functional network topology with behavioral test and molecular biomarkers, through which the curative effects of TSA on miR-30e-induced memory deficit would hopefully be better understood.

## Materials and Methods

### Lentivirus Vector Construction

**FIGURE 1:**
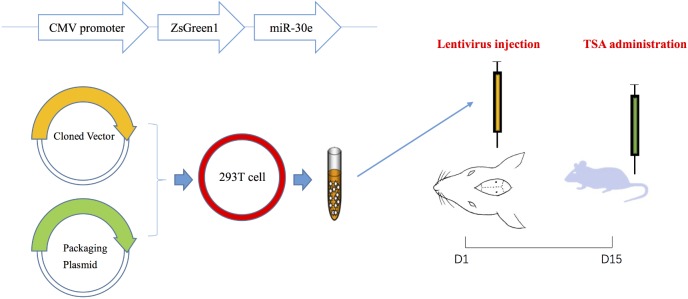
The procedures of lentivirus construction, injection and drug administration.

To deliver the target gene safely and stably into the hippocampus, lentiviral vectors that were replication-deficient and did not leave the site of injection were used to recombine with miR-30e (Xu et al., 2016). Rat pre-miR-30e (Gene ID: 100314153) fragment was amplified by PCR using the following primers: forward 5′-CAACAGAAGGCTCGAGCTGTTGGAGAAGTGGGCATC-3′ and reverse 5′-ATTCTGATCAGGATC CCTCCA AACGAAGAGAGACAGTC-3′, which carried restriction sites for *Bam*HI and *Xho*I, respectively. After PCR cycling and sequence identification, the miR-30e fragment was cut with *Bam*HI and *Xho*I, and then cloned into the PLVX-IRES-ZsGreen1 Expression System (Shanghai SBO Medical Biotechnology Co., Ltd., Shanghai, China). Subsequently, the miR-30e overexpression lentiviral vectors and lentiviral packaging plasmid were mixed and co-transferred into 293T cells. The medium was collected 48 h post-transfection (**Figure 1**). All lentiviral vectors expressed green fluorescent protein (GFP) that helped to measure efficiency of transfection. Additionally, the other lentiviral vectors without carrying miR-30e were also packaged and collected, which were used in sham group and aimed to exclude the influence of lentivirus itself on cognition.

### Animals and Groups

Forty male adult Sprague–Dawley rats were obtained from the Animal center of Military Medical Science Academy, Beijing, China (SCXK-2014-0013). Rats were group-housed in poly propylene cages (475 mm × 345 mm × 200 mm) with *ad libitum* access to food and water. Cages were kept in a temperature (23°C) and 12 h/12 h light/dark-cycle controlled room. Rats were allowed to adapt to the new environment for 7 days before the start of experiments.

Forty rats were randomly arranged into five groups (*n* = 8 each): Rats without surgery + saline treatment (control), empty lentiviral vectors + saline treatment (sham), miR-30e lentiviral vectors + saline treatment (model), miR-30e lentiviral vectors + TSA 10 mL/kg, 1 mg/mL (TSA low), and miR-30e lentiviral vectors + TSA 10 mL/kg, 2 mg/mL (TSA high) (**Table 1**).

**Table 1:** Subdivision of animal groups.

Groups	Bilateral hippocampus transfection	Drug administration via caudal vein
Control	No surgery	Saline (10 mL/kg)
Sham	Lentiviral	Saline (10 mL/kg)
Model	Lentiviral+miR-30e	Saline (10 mL/kg)
TSA low	Lentiviral+miR-30e	TSA (10 mL/kg, 1 mg/mL)
TSA high	Lentiviral+miR-30e	TSA (10 mL/kg, 2 mg/mL)


Model rats were constructed by lentivirus vectors carrying mir-30e into the CA1 region of the hippocampus and continually expressed to produce memory deficit. After anaesthetization by 10% choral hydrate (2 mg/kg), rats were placed on a stereotaxic device (ST-51600; Kopf Instruments, Tujunga, CA, United States). Injection coordinates relative to bregma were as follows: anteroposterior, –4.52 mm; lateral, ±3.2 mm; and vertical, –3.16 mm. Two microliters of lenti-miR-30e infusions were slowly injected into both sides of the hippocampal CA1 region by using a 5-μl 22G microliter syringe (Gaoge Industrial and Trading Co., Ltd. Shanghai, China). The needles were retained in place for 20 min after injections on each side and then very slowly withdrawn to prevent solution backflow. Rats of sham group received lentivirus without carrying miR-30e following the same process to exclude the influence of lentivirus itself on cognition.

Total salvianolic acid was obtained from Tasly Pharmaceutical Co., Ltd. The dose of TSA used in our study was based on data showing that a higher dose (20 mg/mL/kg) in rats can achieve the same plasma concentration in humans. Drugs were administered through the caudal vein for 14 consecutive days, while other groups received equivalent amounts of sterile saline at the same time.

### Behavioral Test

#### Y Maze Test

**FIGURE 2:**
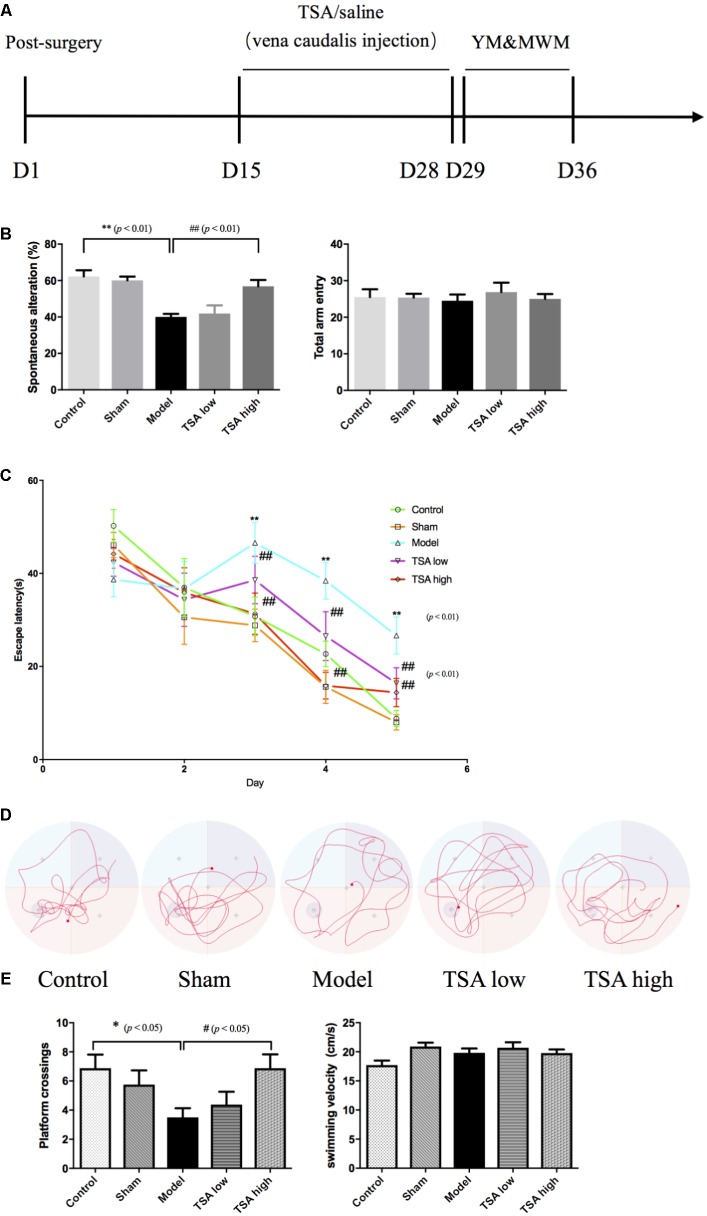
Total salvianolic acid (TSA) improves miR-30e induced learning and memory impairment. **(A)** Timeline of drug administration and behavior test (*n* = 8 per group). After 2 weeks of stereotaxic surgery, rats received a 2-week consecutive drug administration, then initiated behavioral tests. YM, Y maze test; MWM, Morris water maze. **(B)** Spontaneous alteration percent can indicate the working memory of rats in YM, and total arm entry was used to eliminate influences of movement. **(C)** The escape latency refers to the average time needed to locate the platform during the training phase. **(D)** The typical swim paths of each group in the probe phase. **(E)** The platform crossings in probe trail can indicate long-term spatial memory of rats during probe phase of MWM, and average swimming speed was used to eliminate influences of swimming during MWM. Data are shown as the mean ± SEM, ^∗^*p* < 0.05, ^∗∗^*p* < 0.01 (rats v/s control), ^#^*p* < 0.05, ^##^*p* < 0.01 (rats v/s TSA high).

After the 14-day consecutive drug administration, Y maze test was used to examine the rats’ hippocampus-dependent spatial working memory, a form of short-term memory (**Figure 2A**). The Y maze comprises three horizontal arms (100-cm long, 10-cm wide and with a wall that measures 15 cm in height) positioned at 120° to each other. The wall and floor of the maze were painted black. Rats were randomly placed within one arm and could run freely without any restriction for 8 min, during which the sequence and number of each arm entries were recorded manually. An actual alteration was defined as consecutive entries to each arm (such as ABC, BCA, CAB, but not BAB). Percentage of spontaneous alterations was calculated according to the following formula: [(number of alterations)/(total number of arm entries – 2)] × 100 (Kitanaka et al., 2015). The Y maze test was carried out between 14:00 and 17:00 h.

#### Morris Water Maze (MWM)

Long-term spatial memory of rats was evaluated by the MWM task (D’Hooge and De Deyn, 2001). The apparatus is a large round tank (180 cm in diameter and 50 cm in depth) with a removable hidden platform (15 cm in diameter) which is submerged about 3 cm below the surface of water (21 ± 1°C). Markers made of different colors and shapes were placed to give cues for location. During the task, the tank was divided into four quadrants, and the platform was fixed in the middle of the fourth quadrant. First, each rat received four training tasks per day for five consecutive days, and each animal group regard as a training session. During a training session, rats were placed facing the tank wall and then allowed to enter into the water from different directions. After all the rats in a given session finished one direction, they would start the next direction. As each rat was attempting to find the hidden platform, the latency of escape to the hidden platform was recorded if the time was less than 60 s. If a rat failed to find the platform within 60 s, rats will stay at platform for 15 s. Second, probe trail started after training tasks, in which the platform was removed and platform zone cross time was recorded. Parameters of escape latency and platform zone cross time provided information regarding the learning and memory ability, while swimming speed excluded motor disorders. The test was carried out between 10:00 and 14:00 h.

### Resting-State Functional MRI

#### Image Acquisition

**FIGURE 3:**
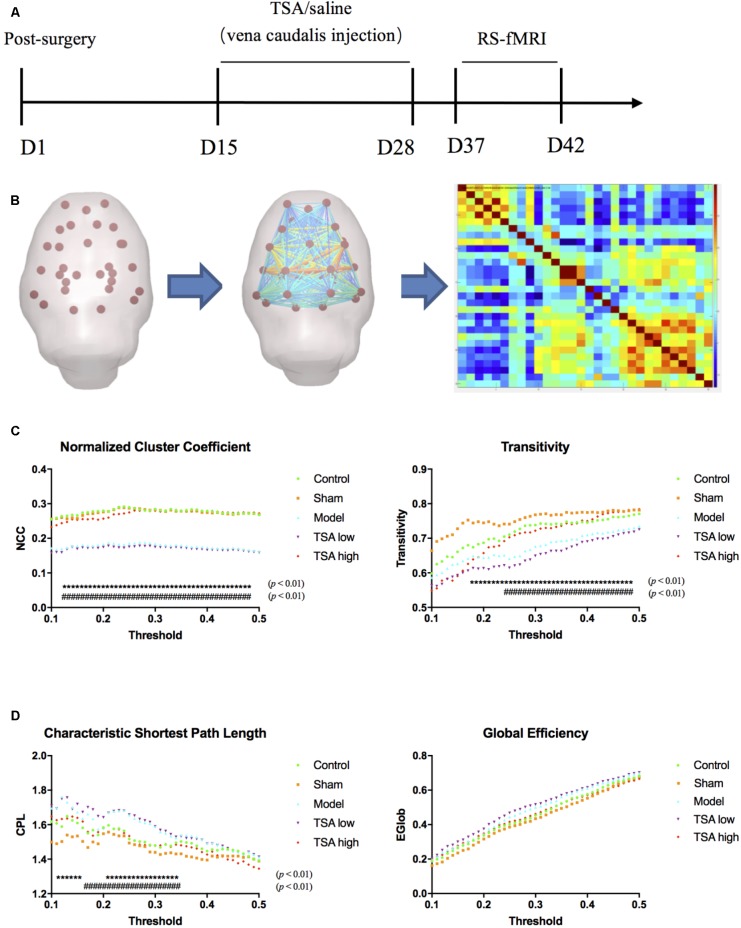
Global measures of brain functional network topology. **(A)** Timelines of BOLD images acquisition. **(B)** Pre-Processed data was interrogated into 30 brain regions of interest (ROI), and correlation matrix was generated. **(C)** Normalized cluster coefficient refers to a normalized version of the mean of the clustering coefficients of all nodes in the network, and transitivity refers to the fraction of triangles around an individual node, which are parameters of functional segregations. **(D)** Characteristic path length (CPL) refers to the average shortest number of weighted edges connecting between any two nodes in the brain, and Global efficiency refers to the grand mean of the inverse distance across all nodes in the brain, which are parameters of functional integration. ^∗^ rats v/s control, # rats v/s TSA high, *p* < 0.01.

All data were obtained using a 3.0T MR scanner (Trivo, Siemens, Germany) with an animal coil (Chenguang Medical Science and Technology, Shanghai, China) (**Figure 3A**). Rats were anesthetized with 10% choral hydrate (2 mg/kg), and then placed on a custom-made plastic bed with the head secured. First, routine T2-weighted image (axial, coronal, sagittal) and three-dimensional T1-weighted structural scan were acquired using the following parameters: matrix size = 68 × 68, field of view = 60 mm, slice thickness = 0.9 mm with a 4.5-mm gap and interleaved, number of slices = 15, echo time = 2.95 ms, repetition time = 1.3 s, and number of volumes = 360. Subsequently, resting-state blood oxygen level-dependent (BOLD) images were scanned using the gradient-echo echo-planar imaging (EPI) sequence using the following parameters: matrix size = 68 × 68, field of view = 40 mm, slice thickness = 1.8 mm with no gap and interleaved, number of slices = 15, echo time = 30 ms, repetition time = 2.3 s, number of volumes = 360.

#### Processing and Analysis

Image preprocessing was performed with Data Processing & Analysis of Brain Imaging (DPABI) V3.0 for Rat Data (Yan et al., 2016). Briefly, all data were converted from DICOM to NIFTI format and the voxel dimensions of all images were scaled by a constant (10) to fit the standard neuroimaging software. Using MRIcro^1^ and DPABI Image Calculator toolbox, all the images were skull stripped manually with brain part masks. After reorientation with the Paxinos rat brain template, a group-specific template was created with structural images to minimize system co-registration error (Yan et al., 2017). Subsequently, the BOLD images were processed following slice timing, motion correction, and spatial normalization.

**FIGURE 4:**
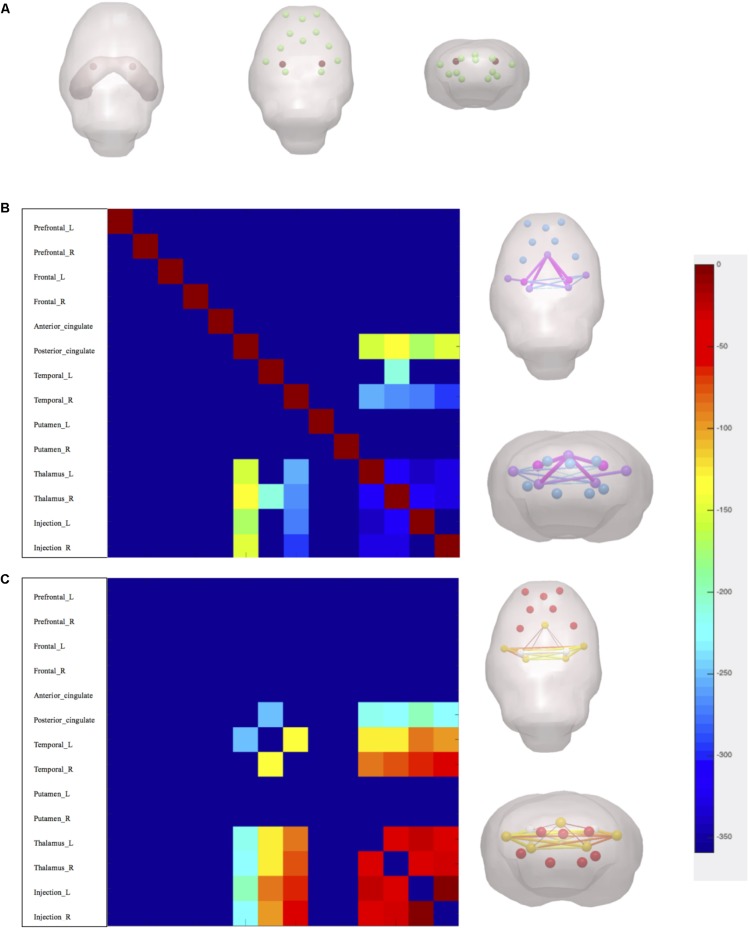
Nodal measures of brain functional network topology. **(A)** Injection sites marks in red and interests of nodes. **(B)** Axial and coronal 3-dimensional-rendered images illustrate statistical difference of edge strength between rats v/s control. **(C)** Axial and coronal 3-dimensional-rendered images illustrate statistical difference of edge strength between rats v/s TSA high. *p* < 0.05, FDR corrected.

After linear detrending and 0.01–0.08-Hz bandpass filter, 30 regions of interest (ROIs) nodes were generated with reference to rat brain atlas (Schwarz et al., 2006). Subsequently, the directed and weighted functional connectivity matrices were constructed using GraphVar Software (Kruschwitz et al., 2015) (**Figure 3B**). The topologic properties of brain functional network were measured at both global and nodal levels with constructed connectivity matrices (Li et al., 2018). At the global level, brain network topologic balance was determined by functional segregation and functional integration. To illustrate functional segregation, we measured normalized cluster coefficients and transitivity, which demonstrated the fraction of triangles around an individual node and a normalized version of the mean of the clustering coefficients of all nodes in the network. For functional integration, characteristic shortest path length (CPL) and global efficiency (EGLOB) were measured, which indicated the average shortest number of weighted edges connecting between any two nodes in the brain and the grand mean of the inverse distance across all nodes in the brain. At the nodal level, we calculated connect strength (edge strength) between CA1 regions (lentivirus-injected sites) and other memory function-related brain regions including the prefrontal cortex, frontal cortex, cingulate, temporal, thalamus, and putamen (**Figure 4A**).

### Immunohistochemistry

Rats (*n* = 6 for each group) were deeply anesthetized with 10% choral hydrate (2 mg/kg), and perfused transcardially with ice-old saline, followed by paraformaldehyde in 0.2M phosphate buffer. The brains were extracted, then post-fixed overnight at 4°C, and transferred into a solution containing 30% sucrose in 4% paraformaldehyde overnight at 4°C for cryoprotection. Sagittal cryostat sections (10 μm) were prepared, and immunohistochemistry was performed using B-cell lymphoma 2 (BCL-2), ubiquitin-conjugating enzyme 9 (UBC9), and Caspase-3 antibodies. The primary antibody was replaced with normal serum in negative controls. The images were captured by Scanscope Digital Pathology Scanning System (Aperio, United States) and analyzed with Cytoplasmic V2 software. Then the gray level of IHC staining results were transformed into optical density (OD).

### Statistical Analysis

All data were expressed as mean ± standard error of the mean (SEM). One-way analysis of variance (ANOVA) was used to analyze the behavioral, global parameters of RS-fMRI and IHC staining data of group differences at *p* < 0.05. The correlations between behavioral test, functional connectivity, and molecular expression level were analyzed by Pearson’s linear correlations. These analyses were carried out by GraphPad Prism Software (San Diego, CA, United States). The edge strength parameters of RS-fMRI were compared between different groups using GraphVar Statistical Toolbox at *p* < 0.05 with false discovery rate (FDR) correction.

## Results

### TSA Attenuates miR-30e Overexpression-Induced Memory Impairment

The spatial working memory was evaluated by spontaneous alteration percent in the Y maze task. Overexpression of miR-30e significantly lowered spontaneous alteration percent of rats v/s control (*p* < 0.01, **Figure 2B**). The long-term spatial learning and memory was estimated by the MWM, in which learning ability was measured by escape latency and long-term memory was measured by platform crossings, respectively. As shown in **Figure 2C**, after miR-30e overexpression, escape latency and the average time needed to locate the platform was significantly prolonged in rats compared with that of controls from the 3rd day to the 5th day during training phase (*p* < 0.01). After the 5-day training, the probe test was carried out by removing the platform from the pool. In the probe test, the results revealed that rats with miR-30e overexpression had lower frequency of platform crossing than the controls (*p* < 0.01, **Figures 2D,E**). These results suggested that miR-30e overexpression impaired both working memory and long-term memory of rats. After administration of TSA, the TSA high group showed more improved spontaneous alteration than miR-30e overexpressed rats in Y maze (*p* < 0.01, **Figure 2B**) and exhibited shorter escape latency and increased platform crossings in the MWM task (*p* < 0.01, **Figure 2C**; *p* < 0.05, **Figure 2E**). However, the TSA low group only had slightly shorter escape latency in MWM. These results indicated that high dose of TSA can significantly ameliorate memory performance of miR-30e overexpressed rats. In addition, the mean swimming speed in MWM and total arm entry in the Y maze were used to exclude the influence of locomotive activity.

### TSA Balances Brain Topologic Connection Network at Global and Nodal Level

At global level of brain network topology, there are four parameters to evaluate functional segregation (normalized cluster coefficient and transitivity) and functional integration (characteristic shortest path length and Global efficiency). After miR-30e overexpression, rats significantly showed decreased normalized cluster coefficient (NCC) and transitivity than the controls (*p* < 0.01, **Figure 3C**) and increased characteristic shortest path length (CPL) than the controls (*p* < 0.01, **Figure 3D**), which may suggest that overexpression of miR-30e can downregulate functional segregation and upregulate functional integration of global network leading to a disturbed global functional network. After TSA treatment, the NCC, transitivity, and CPL of the TSA high group returned to normal levels as compared with miR-30e overexpressed rats (*p* < 0.01, **Figures 3C,D**); and the TSA low group did not show similar significant effects. Therefore, we can conclude that the high dose of TSA effectively ameliorates global network balances which was disturbed by miR-30e overexpression.

At nodal level, edge strength was evaluated between nodes including the prefrontal cortex, frontal cortex, cingulate, temporal lobes, thalamus, putamen, and hippocampus CA1 regions. The results showed that after miR-30e overexpression edge strength of rats decreased between CA1 and posterior cingulate, thalamus, and temporal lobes as compared with control (*p* < 0.05, FDR corrected, **Figure 4B**). After administration of TSA, high dose of TSA significantly increased edge strength between CA1 and thalamus, temporal of rats (*p* < 0.05, FDR corrected, **Figure 4C**). It can be inferred that high dose of TSA can also improve nodal level connection of brain network topology.

### TSA Inhibits miR-30e-Induced Neuronal Apoptosis in Hippocampus CA1

**FIGURE 5:**
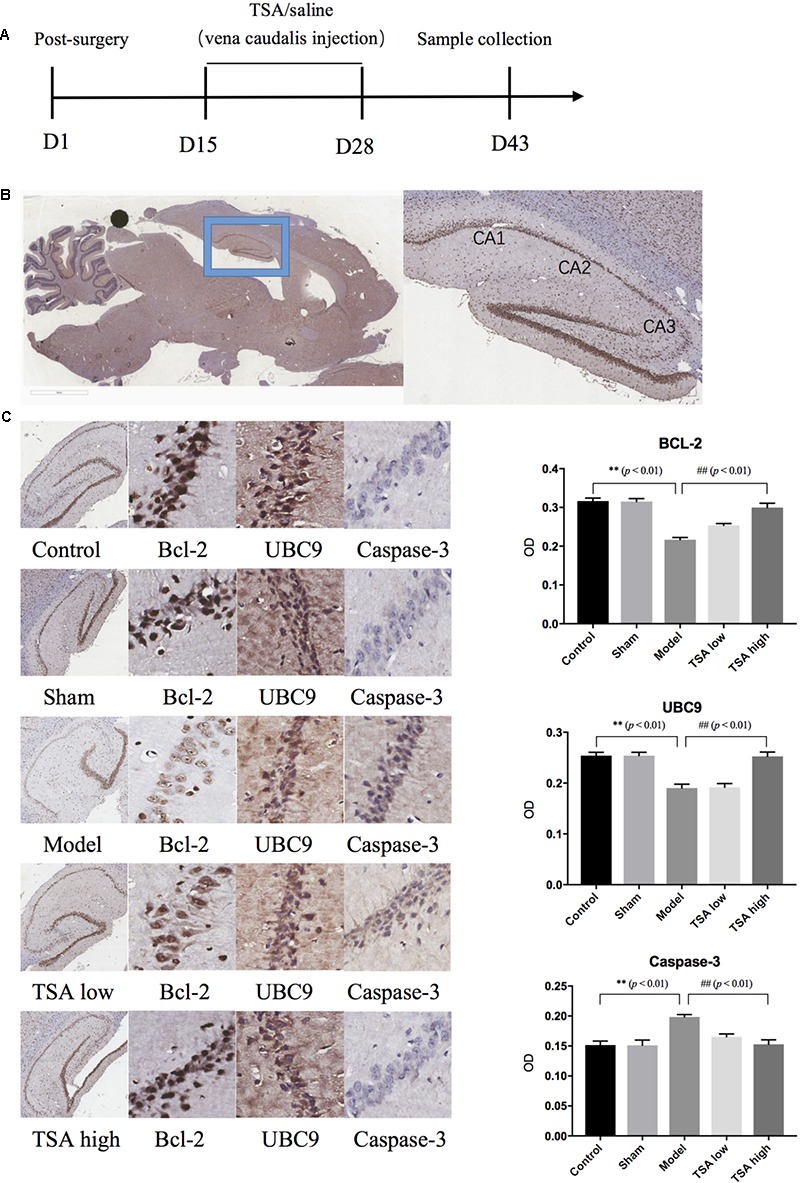
Total salvianolic acid inhibits neuron apoptosis induced by miR-30e. **(A)** Timeline of immunohistochemistry sample collection. **(B)** Sagittal section of rat brain and sub-regions of hippocampus. **(C)** Immunohistochemistry staining of Bcl-2, UBC9, and Caspase-3 in each group. Data are shown as the mean ± SEM, ^∗^*p* < 0.05, ^∗∗^*p* < 0.01 (rats v/s control), ^#^*p* < 0.05, ^##^*p* < 0.01 (rats v/s TSA high).

**Figure 5** shows the results of immunohistochemical analysis of expression levels of apoptosis-related proteins. After miR-30e overexpression, the protein expression levels of Bcl-2 and UBC9 were significantly reduced in rats compared with controls (*p* < 0.01, **Figure 5C**), and the levels of Caspase-3 showed an opposite result that were significantly increased (*p* < 0.01, **Figure 5C**). Following TSA treatment, the protein expression levels of BCL-2, UBC9, and Caspase-3 returned to normal in the TSA high group and were not significantly different from those of the control (*p* < 0.01, **Figure 5C**). These results likely illustrate that high dose of TSA inhibits miR-30e induced apoptosis of neurons.

### Functional Network Topology Correlates With Behavioral Performance and Molecular Alterations

**FIGURE 6:**
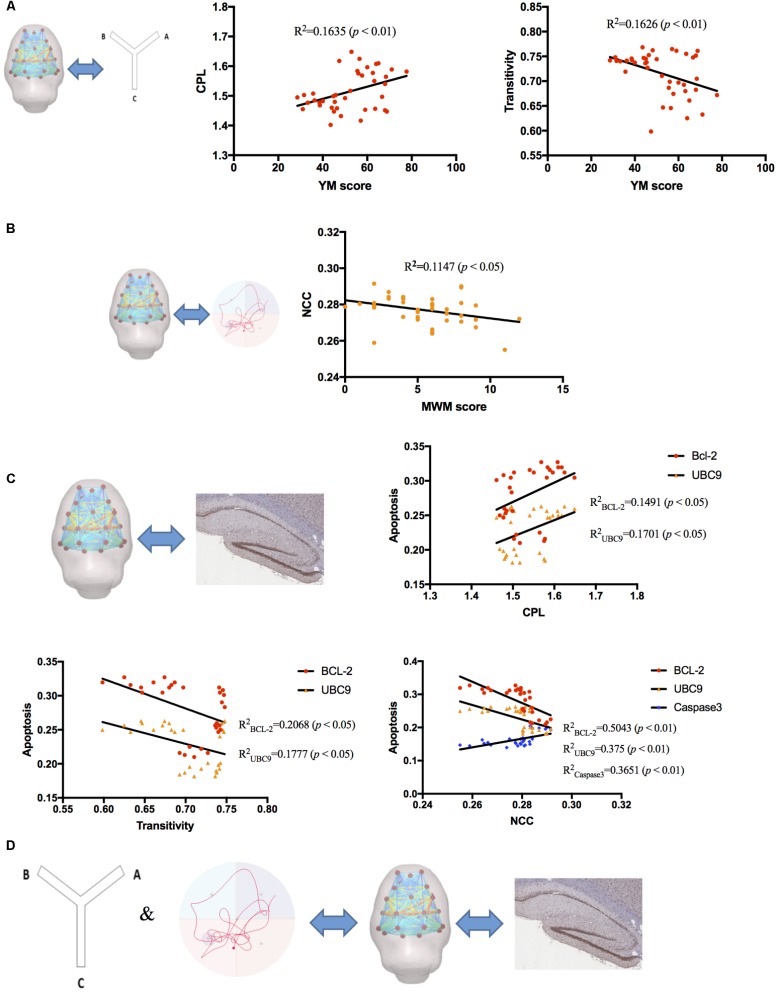
The correlation analysis between RS-fMRI data, behavioral test, and molecular alterations. **(A)** YM scores positively correlated with CPL (*R*^2^ = 0.1635, *p* < 0.01) and negatively correlated with transitivity (*R*^2^= 0.1626, *p* < 0.01). **(B)** MWM scores negatively correlated with NCC (*R*^2^= 0.1147, *p* < 0.05). **(C)** CPL positively correlated with BCL-2 and UBC9 (*R*^2^_BCL-2_ = 0.1491, *R*^2^_UBC9_ = 0.1701, *p* < 0.05); Transitivity negatively correlated with BCL-2 and UBC9 (*R*^2^_BCL-2_ = 0.2068, *R*^2^_UBC9_ = 0.1777, *p* < 0.05); NCC negatively correlated with BCL-2 and UBC9 and positively correlated with Caspase-3 (*R*^2^_BCL-2_ = 0.5043, *R*^2^_UBC9_ = 0.375, *R*^2^_caspase3_ = 0.3651, *p* < 0.01). **(D)** Global brain functional network topology may be an intermediate biomarker between behavioral changes and molecular alterations.

Correlation analyses were measured between global parameters and behavioral scores, global parameters and protein expression level, respectively, by considering all subjects as a group. Our results showed that YM scores positively correlated with CPL (*R*^2^ = 0.1635, *p* < 0.01) and negatively correlated with transitivity (*R*^2^= 0.1626, *p* < 0.01) (**Figure 6A**), while MWM scores negatively correlated with NCC (*R*^2^ = 0.1147, *p* < 0.05) (**Figure 6B**). The correlation between global parameters and molecular alterations exhibited that CPL positively correlated with BCL-2 and UBC9 (*R*^2^_BCL-2_ = 0.1491, *R*^2^_UBC9_ = 0.1701, *p* < 0.05); Transitivity negatively correlated with BCL-2 and UBC9 (*R*^2^_BCL-2_ = 0.2068, *R*^2^_UBC9_ = 0.1777, *p* < 0.05); NCC negatively correlated with BCL-2 and UBC9 and positively correlated with Caspase-3 (*R*^2^_BCL-2_ = 0.5043, *R*^2^_UBC9_ = 0.375, *R*^2^_caspase3_ = 0.3651, *p* < 0.01) (**Figure 6C**). These results demonstrated that correlations between global parameters and behavioral scores, global parameters and protein expression level had similar tendencies which may indicate that Global brain functional network topology may be an intermediate biomarker between behavioral changes and molecular alterations (**Figure 6D**).

## Discussion

Memory is a dynamic brain function during which different brain regions are functionally collaborated as in a network (Beason-Held et al., 2017). The connections between each region are not random but are organized as a balance between segregation and integration (Vecchio et al., 2015). To analyze the topologic properties of brain network, graph theory was regarded as an effective measure that investigates the relationship of nodes and edges of a network by using several parameters (Hadley et al., 2016). In the present study, we used RS-fMRI data to constructed a functional network and analyzed topologic properties by both global and nodal parameters.

Recent research has indicated that global parameters of brain network topology, which aims to provide a full understanding of the segregation and integration of spatiotemporal information across networks, can be potential biomarkers of neuropsychiatric disorders (Deco and Kringelbach, 2014), for which may represent an early deleterious consequence of synaptic pathologies (Vecchio et al., 2015). Functional segregation in the brain is the ability for specialized processing to occur within densely interconnected groups of brain regions; thus, according to the definition, a higher cluster coefficient and transitivity network represent greater functional segregation. Also, functional integration is defined as the ability to rapidly combine specialized information from distributed brain regions; hence, a shorter characteristic shortest path length and global efficiency network implies greater functional integration (Rubinov and Sporns, 2010). Our results indicated that miR-30e overexpression caused a lowered functional segregation and excess functional integration which may lead to a poor global efficiency in rats, and that a high dose of TSA may have potential therapeutic effects to improve the global functional network.

Brain network topology is also an effective tool to evaluate the connection strength of the default mode network (DMN) at the nodal level (Akiki et al., 2018). With the development of RS-fMRI, more and more functional imaging data has indicated that different brain regions can be organized into several networks to carry out different brain functions (Barkhof et al., 2014) and DMN was one of the first functional MRI networks to investigate memory dysfunction in aging (Meskaldji et al., 2016). DMN includes several brain regions such as posterior cingulate (Leech and Sharp, 2014; Wu et al., 2014), temporal lobe (Jeneson and Squire, 2012), and thalamus (de Bourbon-Teles et al., 2014), and these regions were proved to play an important role in learning and memory. In the present study, we chose the bilateral prefrontal cortex, frontal cortex, cingulate, temporal, thalamus, putamen, and hippocampus CA1 regions as nodes and analyzed connections with the topologic edge strength parameter. Our data demonstrated that mir-30e overexpression decreased nodal strength between CA1 and the posterior cingulate, temporal lobe, and thalamus, and high dose of TSA can revert these changes to the normal level, which may be another potential mechanism of TSA’s therapeutic effect.

The behavioral changes in rats have been confirmed by RS-fMRI that had a linear correlation with functional topology networks (Nasrallah et al., 2016). Therefore, we chose the Y maze and MWM tasks, which are the most commonly used behavioral tests on rats’ memory performance (D’Hooge and De Deyn, 2001; Kim et al., 2006), to explore the effects of miR-30e overexpression and TSA treatment on rat behavior. Our results were consistent with those of previous studies wherein continuous overexpression of miR-30e in CA1 led to memory impairment (Xu et al., 2015). In addition, we found that high dose of TSA significantly ameliorates memory functions in rats. Correlation analyses of our results also showed similar findings that behavioral results were correlated to functional topology networks. Taken together, our results illustrated that inhibiting miR-30e overexpression in CA1 neurons improves global network efficiency, which can lead to better behavioral outcomes.

It has been demonstrated that disturbance of hippocampus CA1 regions induce a remarkable impairment in spatial learning and memory (Luo et al., 2015; Yuan et al., 2016). Previous studies indicated that hippocampi CA1 disturbance induced by miR-30e overexpression may associated with increased neuron apoptosis which caused by decreasing the expression level of BCl-2 and UBC9 (Xu et al., 2015). In addition, miR-30e also targeted expression of caspase-3 (Sohn et al., 2016). To achieve cognitive protection of hippocampi CA1, existing investigations showed that it is a helpful way through inhibiting caspase-3 and promoting the BCl-2 expression in the CA1 region neurons of the hippocampus (Ma et al., 2011; Qi et al., 2016). In the present study, our data showed decreased expression levels of BCL-2, UBC9, and increased levels of caspase-3 in the miR-30e overexpression group, similar to previous studies, and indicated that neuron apoptosis increased in CA1 regions of hippocampus where lentiviral vectors were injected. After TSA treatment, the expression level of BCl-2, UBC9, and caspase-3 reverted to normal standard of control, which shows that TSA can suppress the effects of miR-30e overexpression and reduce neuron apoptosis of the CA1 region. Further, our correlation analyses revealed that functional segregation and integration can be an intermediate marker to link molecular alterations and behavioral performances.

A major limitation of this study is the use of the 3.0T MRI scanner that could not provide sufficient details of the brain regions as the 7.0T scanner. Although apoptosis-related molecular mechanisms of TSA therapeutic effects have been demonstrated in the present study, several other cell signaling pathways related to memory functions and neuroprotection still need to be evaluated and understood.

## Ethics Statement

This study was carried out in accordance with the recommendations of the National Institute of Health Guide for the care and use of laboratory animals. The protocol was approved by the Animal Ethical Committee of Shanxi Medical University.

## Author Contributions

YX and C-GY designed and supervised the study. QL, LW, and X-YL drafted the manuscript. QL and LW carried out the experimental procedures. XC and BL undertook the statistical analyses and reviewed the literature. LC participated in data processing.

## Conflict of Interest Statement

The authors declare that the research was conducted in the absence of any commercial or financial relationships that could be construed as a potential conflict of interest.

## Footnotes

1 http://www.mccauslandcenter.sc.edu/crnl/MRIcro
